# A Kinase-Phosphatase Switch Transduces Environmental Information into a Bacterial Cell Cycle Circuit

**DOI:** 10.1371/journal.pgen.1006522

**Published:** 2016-12-12

**Authors:** Kristina Heinrich, Patrick Sobetzko, Kristina Jonas

**Affiliations:** 1 Science for Life Laboratory, Department of Molecular Biosciences, The Wenner-Gren Institute, Stockholm University, Stockholm, Sweden; 2 LOEWE Center for Synthetic Microbiology (SYNMIKRO), Philipps University Marburg, Marburg, Germany; University of Geneva Medical School, SWITZERLAND

## Abstract

The bacterial cell cycle has been extensively studied under standard growth conditions. How it is modulated in response to environmental changes remains poorly understood. Here, we demonstrate that the freshwater bacterium *Caulobacter crescentus* blocks cell division and grows to filamentous cells in response to stress conditions affecting the cell membrane. Our data suggest that stress switches the membrane-bound cell cycle kinase CckA to its phosphatase mode, leading to the rapid dephosphorylation, inactivation and proteolysis of the master cell cycle regulator CtrA. The clearance of CtrA results in downregulation of division and morphogenesis genes and consequently a cell division block. Upon shift to non-stress conditions, cells quickly restart cell division and return to normal cell size. Our data indicate that the temporary inhibition of cell division through the regulated inactivation of CtrA constitutes a growth advantage under stress. Taken together, our work reveals a new mechanism that allows bacteria to alter their mode of proliferation in response to environmental cues by controlling the activity of a master cell cycle transcription factor. Furthermore, our results highlight the role of a bifunctional kinase in this process that integrates the cell cycle with environmental information.

## Author Summary

Free-living bacteria are frequently exposed to various environmental stress conditions. To survive under such adverse conditions, cells must induce pathways that prevent and alleviate cellular damages, but they must also adjust their cell cycle to guarantee cellular integrity. It has long been observed that various bacteria transform into filamentous cells under certain conditions in nature, indicating that they dynamically modulate cell division and the cell cycle in response to environmental cues. The molecular bases that allow bacteria to regulate cell division in response to fluctuating environmental conditions remain poorly understood. Here, we describe a new mechanism by which *Caulobacter crescentus* blocks division and transforms into filamentous cells under stress. We find that the observed cell division block depends on precise regulation of the key cell cycle regulator CtrA. Under optimal conditions, the membrane-bound cell cycle kinase CckA activates CtrA in response to spatiotemporal cues to induce expression of genes required for cell division. Our data suggest that external stress triggers CckA to dephosphorylate and inactivate CtrA, thus ensuring the downregulation of CtrA-regulated functions, including cell division. Given that CckA and CtrA are highly conserved among alphaproteobacteria, the mechanism found here, might operate in diverse bacteria, including those that are medically and agriculturally relevant.

## Introduction

The bacterial cell cycle has been studied extensively in the past. Genetics, biochemistry and more recently, advanced microscopy techniques have provided important insight into the processes of DNA replication, chromosome segregation and cell division, and numerous regulatory mechanisms have been identified that precisely coordinate these processes in time and space. Most of this research has focused on cell cycle regulation under standard and stable laboratory growth conditions. However, in nature bacteria are exposed to drastic environmental changes, where they have to constantly adjust their growth rate and mode of proliferation [1,2]. It has frequently been reported that various bacteria transform into multi-chromosome containing filaments in response to certain environmental conditions [2–4], indicating that bacteria dynamically modulate cell division and the cell cycle in response to environmental cues. Nevertheless, the precise mechanisms transducing environmental information into the cell division machinery and how these mechanisms help cells to survive under adverse conditions are not well understood.

Cell cycle regulation has been studied in several model bacteria. One prominent example is the asymmetrically dividing alphaproteobacterium *Caulobacter crescentus*, a freshwater bacterium that mainly occurs in oligotrophic aquatic environments, but also in organically rich environments such as wastewater [5]. The *Caulobacter* cell cycle is characterized by asymmetric cell division and well-defined, morphologically distinct cell cycle phases, offering the possibility to examine cell cycle progression with high spatial and temporal resolution. Past work has identified a suite of key regulatory proteins required for cell cycle progression and important progress has been made in understanding how these factors are wired in higher-ordered circuits to drive cell cycle progression under optimal conditions [6,7]. However, how the *Caulobacter* cell cycle is modulated in response to environmental changes is only at the beginning of being explored.

One major cell cycle regulator is the conserved response regulator CtrA, which regulates the transcription of nearly 100 genes involved in cell division, cell cycle regulation and morphogenesis [8,9]. By binding to the origin of DNA replication CtrA also serves as a negative regulator of DNA replication initiation [10]. CtrA activity is strictly regulated and oscillates in a cell cycle-dependent manner [11]. In G1-phase CtrA is active and represses the origin [10]. At the G1-to-S transition it is inactivated and rapidly proteolysed allowing DNA replication to initiate [12,13]. During S-phase, active CtrA accumulates again to induce the expression of cell division and morphogenesis genes that are required to complete the cell cycle by cell division [9]. The oscillations of CtrA depend on its precise regulation by the CckA-ChpT phosphorelay, which is comprised of the polarly localized bifunctional histidine kinase CckA and the phosphotransferase ChpT [14,15]. In response to spatiotemporal cues, CckA phosphorylates CtrA via ChpT, resulting in CtrA activation. Reversal of the phosphorelay leads to CtrA dephosphorylation and hence its inactivation [15,16]. CtrA inactivation is tightly coupled to its degradation by the ClpXP protease that depends on CpdR, an adaptor protein, whose activity also depends on CckA-ChpT [17]. In contrast to CtrA, CpdR is only active when dephosphorylated [17,18]. Thus, under conditions when CtrA is inactivated, it is also targeted for degradation by ClpXP and CpdR. In addition to CpdR, the cell-cycle dependent degradation of CtrA involves the adaptors RcdA and PopA that promote CtrA proteolysis by ClpXP in a second messenger and phosphorylation dependent manner [19–21].

Recent work has provided important insight into the mechanisms regulating the CckA kinase in a cell cycle-dependent manner. It was shown that protein interactions with the upstream regulators DivL and DivK at the cell poles allow CckA to switch between kinase and phosphatase activity [22,23]. In addition, CckA activity is also modulated by the second messenger cyclic diguanylate (c-di-GMP) [24]. This molecule, which accumulates at the G1-S transition [25], directly binds CckA and stimulates its phosphatase activity, and thus CtrA inactivation and proteolysis [24]. As a transmembrane protein CckA may also directly respond to external signals, as typical for most other histidine kinases that have important sensing functions and directly transduce environmental information into cellular responses. However, external conditions that influence CckA activity have not been identified until now.

The present study started with a systematic analysis of the effects of different stress conditions on *C*. *crescentus* cell cycle progression. This analysis revealed that under certain conditions *Caulobacter* specifically blocks cell division and grows to filamentous cells. Interestingly, the stress-induced cell filamentation that we observed is not mediated by induction of small cell division inhibitors, as previously described for conditions inducing DNA damage [1,26,27]. Instead, we found that stress leads to rapid downregulation of CtrA. Our data suggest that the stress-induced regulation of CtrA stems from stimulation of CckA phosphatase activity and that it provides a growth advantage under stress.

## Results

### Different stress conditions affect the *C*. *crescentus* cell cycle at different stages

**Fig 1:**
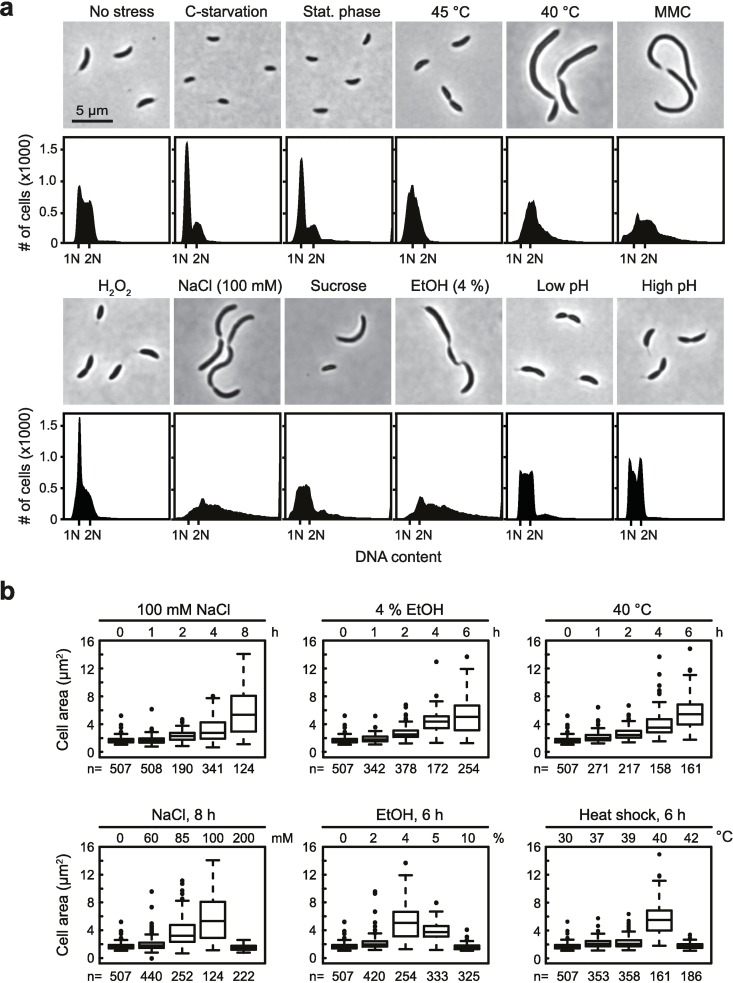
Different stress conditions affect the *C*. *crescentus* cell cycle at different stages. (a) Phase contrast microscopy images and flow cytometry profiles showing cell cycle phenotypes of wild type *C*. *crescentus* under different stress conditions. Fluorescence that equals one (1N) or two (2N) fully replicated chromosomes is indicated in each flow cytometry profile. Data of exponentially growing cells (no stress) are shown for comparison. Stress conditions were chosen at concentrations and time points when cells were still viable but growth rate was slowed (see also S2 Fig) or, in the case of stationary phase and carbon starvation, when growth was arrested. Stationary phase was induced by continuous growth for 48 hours, carbon starvation was induced by growing cells in M2 0.02% glucose for 8 hours. Heat shock data are shown for cultures that have been exposed to 45°C for one hour or to 40°C for 6 hours. DNA damage was induced by treating cells for 6 hours with 3 μg/ml mitomycin C (MMC), oxidative stress by growing cells in the presence of 0.1 mM H_2_O_2_ for 1 hour. For the NaCl, sucrose and ethanol stress conditions data are shown for cells that were treated with 100 mM, 200 mM or 4%, respectively, for 6 hours. Phenotypes at low pH (pH 4.9) and high pH (pH 9.1) were monitored after 1 hour. (b) Box plots showing changes in cell area as a response to treatment with NaCl, EtOH or mild heat shock at different time points (upper panels) or at different concentrations / temperatures (lower panels). The number (n) of analyzed cells is indicated below each plot.

To analyze the consequences of different stress conditions on cell cycle progression in *C*. *crescentus*, we challenged bacterial cultures with carbon starvation, stationary phase, heat shock, DNA damage, oxidative stress, salt stress, sucrose stress, ethanol stress and changes in pH while monitoring changes in cell morphology and chromosome content using phase-contrast microscopy and flow cytometry, respectively. As previously shown, carbon starvation, growth in stationary phase and acute heat shock lead to a block of DNA replication initiation and a G1-arrest while cell size is largely maintained (Fig 1A) [28–30]. Interestingly, several other stress conditions caused *C*. *crescentus* cells to respond in a clearly different manner. Most conspicuously, upon treatment with 100 mM NaCl or 4% ethanol (EtOH) cells transformed into long filaments and accumulated multiple chromosomes (Fig 1A). Consistent with the flow cytometry data, we found by using a fluorescent repressor-operator system (FROS), which fluorescently marks the origins of replication [31], that the elongated cells contained multiple well-segregated origins (S1 Fig), demonstrating that cells continue to undergo DNA replication, chromosome segregation and cellular growth under these conditions, but that they stop dividing. Cells treated with NaCl or ethanol increased to up to eight to ten-fold of their original length and often accumulated six to seven chromosomes within eight hours, suggesting that growth and the cell cycle continued for three to four doubling times in the absence of division. The effects on cell division were observed within two hours of NaCl or EtOH treatment, however the phenotype became more pronounced over time (Fig 1B). We also observed that the filamentous phenotype only occurred in a narrow range of NaCl or EtOH concentrations (Fig 1B), in which cells are still viable and able to grow (S2 Fig).

Similar to the phenotypic response observed upon NaCl or EtOH treatment, we observed that incubation at 40°C also caused cell elongation. At this temperature *C*. *crescentus* is still able to grow and to replicate its DNA, whereas temperatures above 40°C lead to a growth and DNA replication arrest (Fig 1, S2 Fig). The chromosome content was not as strongly increased at 40°C as under the EtOH and NaCl stress conditions.

We did not observe significant changes in cell morphology or chromosome content when cultivating *C*. *crescentus* at different acidic or alkaline pHs (Fig 1A). Treatment of cells with sublethal doses of H_2_O_2_ led to an increased proportion of cells (54.1%) with a DNA content that equals a single chromosome compared to the non-stress condition (38.2%) (Fig 1A), suggesting that under oxidative stress many cells arrest in G1-phase, similar to starvation conditions, stationary phase and acute heat shock.

**Fig 2:**
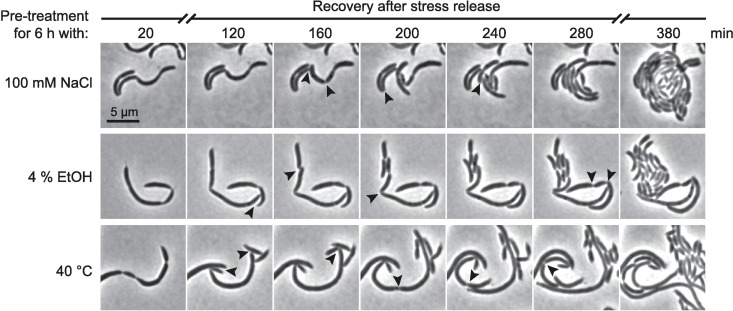
Stress-treated cells resume cell division upon shift to non-stress conditions. The time-lapse images show the recovery of cells following stress treatment. Cells were treated for 6 hours with 100 mM NaCl, 4% EtOH or 40°C, respectively, and transferred to a plain PYE agarose pad to monitor the recovery process of single cells over time. Arrow heads depict cell division sites that give rise to the first generation of daughter cells from a filamentous cell.

To assess whether cell filamentation in response to salt, ethanol or mild heat shock stress is reversible, we followed the fate of stress-treated filamentous cells by time-lapse microscopy upon shifting them to non-stress conditions. Filamentous cells from each of the three conditions were able to resume growth and to initiate cell division at multiple sites shortly after release to fresh growth medium with first cell division events occurring within two hours (Fig 2). Within six hours most bacteria were able to propagate and divide normally yielding typically sized *Caulobacter* daughter cells. Notably, a fraction of daughter cells that arose from the filamentous cells maintained elongated until the end of our time-lapse study, indicating that these cells were severely damaged.

Altogether our data demonstrate that *C*. *crescentus* changes its morphology and cell cycle status in response to changing external conditions. Different stress conditions affect the cell cycle at different stages, either by transiently blocking DNA replication initiation and cellular growth or by delaying cell division.

### Filamentation is not induced by the SOS response or misregulation of FtsZ

**Fig 3:**
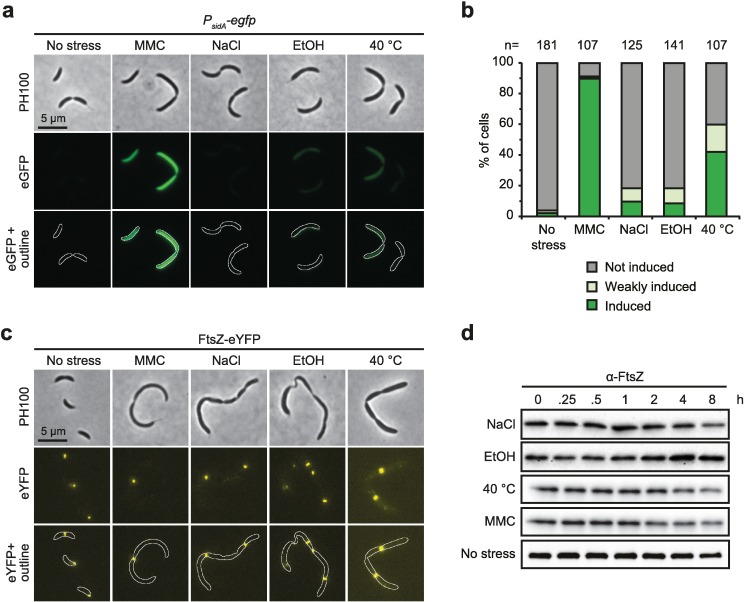
Filamentation is not induced by the SOS response or by an inability to accumulate or localize FtsZ. (a) Phase contrast and fluorescence images of a *P*_*sidA*_*-egfp* reporter strain [26] after treatment for two hours with 3 μg/ml mitomycin C (MMC, positive control), 100 mM NaCl, 4% EtOH or 40°C. (b) Quantification of the results shown in a. Cells with low fluorescence intensity were classified as weakly induced. The number (n) of analyzed cells is indicated above each bar. See also S3 Fig for quantifications using a plate reader. (c) Phase contrast and fluorescence images of cells expressing *ftsZ-eyfp*. Cells were treated for six hours with the respective stress condition. Expression of *ftsZ-eyfp* was induced with 500 μM vanillate two hours prior imaging. (d) Western blots of FtsZ steady-state levels in wild type cells under the indicated stress conditions.

The filamentous phenotype of cells treated with salt, ethanol and mild heat shock is similar to the phenotype of cells treated with DNA damaging agents such as mitomycin C (Fig 1A) [26]. Previously, it has been shown that in *C*. *crescentus* and other bacteria DNA damage induces the expression of small division inhibitors, which directly interfere with components of the cell division apparatus and thereby block the process of cell division in response to DNA damage [1,26,27]. To test whether the transient block of cell division upon treatment with NaCl, EtOH or increased temperature is due to induction of the SOS response we monitored the promoter activity of the *sidA* gene, which encodes the SOS induced division inhibitor in *C*. *crescentus* [26]. In contrast to mitomycin C treatment, which caused strong induction of *P*_*sidA*_*-gfp* within two hours, the reporter was not turned on upon exposure to NaCl or EtOH stress (Fig 3A and 3B, S3 Fig). A mild induction in a subpopulation of cells was observed at 40°C, which was however clearly lower compared to mitomycin treatment. These data show that the filamentous phenotype induced by salt stress, ethanol stress or mild heat shock does not appear to depend on the SOS response and SidA.

Another previous study reported that a subpopulation of *C*. *crescentus* cells form helical filaments during long-term growth in stationary phase [32]. It was shown that in these filaments the level of the major cell division protein FtsZ was strongly reduced [32]. Therefore we wanted to test if FtsZ abundance and localization was affected under NaCl stress, EtOH stress and mild heat shock. However, Western Blot analysis showed that FtsZ protein abundance was not significantly altered within eight hours of stress treatment (Fig 3D). Furthermore, using a strain expressing *ftsZ-eYFP* showed that FtsZ still localized in distinct foci along the length of the cells (Fig 3C), suggesting that the observed block of cell division is not caused by a failure of FtsZ to localize to division sites.

### The regulon of the master cell cycle regulator CtrA is strongly affected during salt and ethanol stress

To better understand the molecular basis underlying the observed stress-induced morphological changes, we analyzed global changes in gene expression by RNA-sequencing (RNA-seq). We focused on EtOH and NaCl stress as the effect on cell division was most pronounced under these conditions.

**Fig 4:**
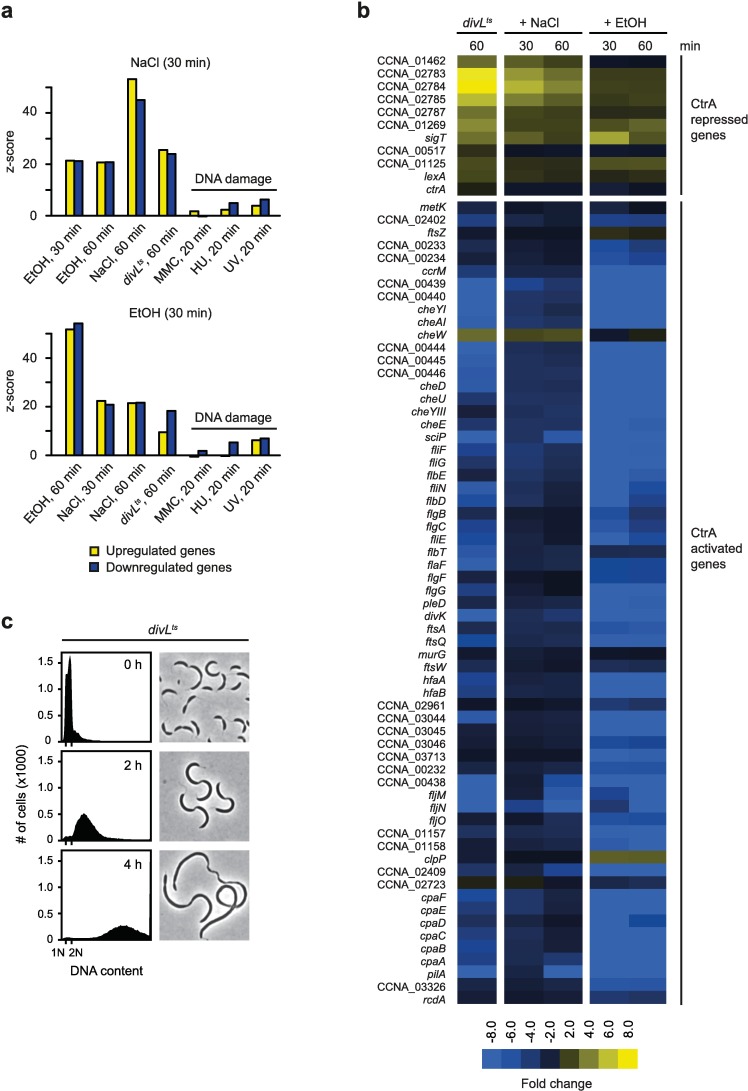
Salt and ethanol stress affect the regulon of the cell cycle master regulator CtrA. (a) Comparison of RNA-seq data from the NaCl (upper graph) and EtOH (lower graph) stress condition with transcriptional data from other conditions. Z-scores indicate the level of similarity between two data sets that were compared. Z-scores for upregulated genes are shown in yellow, z-scores for downregulated genes are shown in blue. EtOH and NaCl conditions were compared with each other, with *divL*^*ts*^ data and with previously published DNA damage microarray data [26]. (b) RNA-seq data showing transcriptional changes of CtrA-regulated genes after DivL inactivation (*divL*^*ts*^), treatment with 100 mM NaCl or treatment with 4% EtOH. (c) Flow cytometry profiles and phase contrast images of a *divL*^*ts*^ mutant before (0 hours) and after (2 and 4 hours) cultivation at the restrictive temperature (37°C).

Treatment with 100 mM NaCl for 60 minutes resulted in a >2-fold induction of 472 genes (11.6%) and a >2-fold downregulation of 282 genes (6.9%). In the case of EtOH stress, 570 genes were >2-fold upregulated (14.0%) and 489 genes (12.0%) were >2-fold downregulated after 60 minutes. Statistical analysis revealed that in each case, the gene expression profile after 60 minutes was highly similar to the profile obtained after 30 minutes of stress treatment with significant scores (z-scores) greater than 40 (Fig 4A). We also observed a strong overlap when comparing the gene expression data sets from the NaCl and EtOH conditions to each other for both upregulated and downregulated genes (z-scores >20), suggesting that salt and ethanol stress result in similar changes in gene expression. Consistent with our data obtained with the *P*_*sidA*_*-gfp* reporter, the SOS regulon was not strongly induced under ethanol and salt stress (Fig 4A).

Interestingly, among the most downregulated genes were many involved in flagella biosynthesis, pili biogenesis, chemotaxis, and cell cycle progression (Fig 4B). In *C*. *crescentus*, these genes are under the direct control of the master cell cycle regulator CtrA [8]. Consistently, we found that gene expression changes in a *divL*^*ts*^ mutant, which upon shift to 37°C results in loss of CtrA function [22,33], were similar to the gene expression changes induced by salt and ethanol stress in wild type cells (Fig 4A and 4B). Genes that were downregulated in *divL*^*ts*^ cells, for example flagella, pili and chemotaxis genes, cell cycle regulators (*ccrM*, *sciP*, *divK*) or cell division genes (*ftsA*, *ftsQ*, *ftsW*) also showed significant downregulation in response to NaCl or EtOH treatment. By contrast, CtrA repressed genes, which were upregulated in the *divL*^*ts*^ mutant also showed upregulation in the NaCl treated and EtOH treated cultures. These data demonstrate that the regulon of CtrA is strongly influenced in response to salt and ethanol stress, suggesting that CtrA activity is affected under these conditions. Loss of CtrA function results in strong chromosome accumulation and cell filamentation [13,22], phenotypes similar to those observed during salt and ethanol stress (Fig 4C).

### CtrA abundance is changed by altered rates of proteolysis

**Fig 5:**
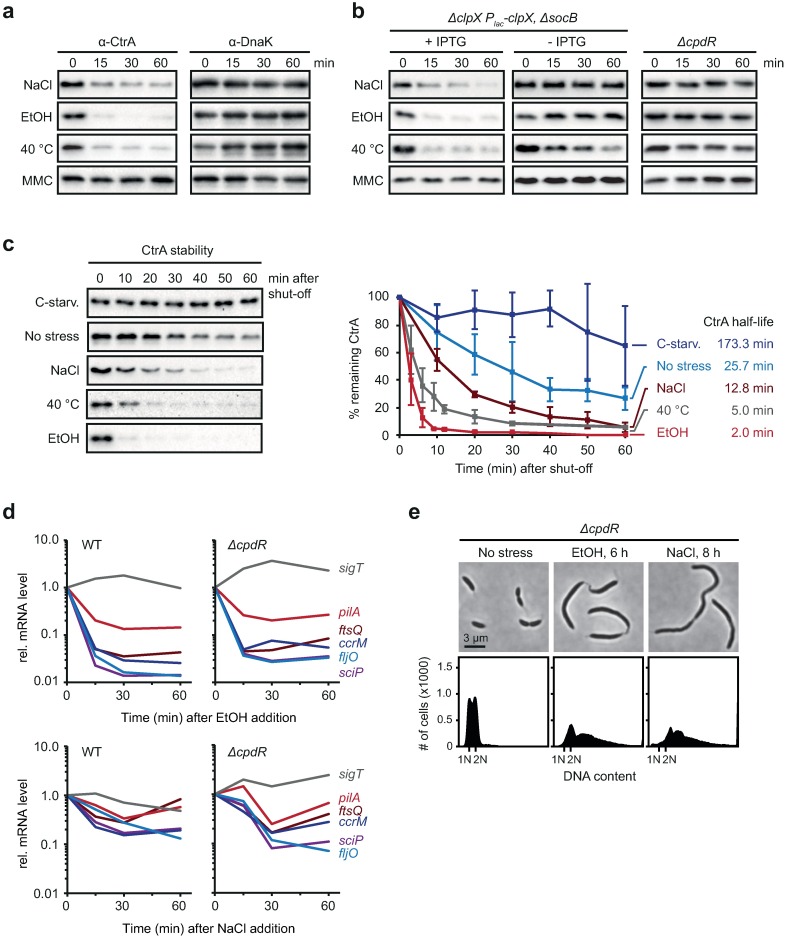
Stress induces the proteolysis and inactivation of CtrA. (a) Western blots showing changes in CtrA steady-state levels following treatment of cultures with NaCl (100 mM), EtOH (4%), heat shock (40°C) or MMC (3 μg/ml). DnaK levels are shown as a control. (b) Steady-state levels of CtrA upon stress treatment in a ClpX depletion strain and a *cpdR* deletion strain. The ClpX depletion strain contains a deletion of *socB* bypassing the lethal effect of ClpX depletion [54]. Data for this strain are shown under non-depleting (+ IPTG) and ClpX-depleting conditions (- IPTG). (c) *In vivo* degradation assays showing CtrA stability in the absence of stress (no stress), during carbon starvation (5 h after shifting cells from M2G to M2 0.02% glucose) and after five minutes of treatment with 100 mM NaCl, 40°C or 4% EtOH. Chloramphenicol was added to shut-off protein synthesis; remaining CtrA protein levels were monitored every ten minutes by Western blotting. The graph shows results from quantifications of band intensities; averages of at least two independent replicates are shown with standard deviations. CtrA half-lives under the different conditions are indicated. See also S4 Fig for additional time points for the EtOH and 40°C conditions. (d) Changes in transcript levels of selected CtrA-regulated genes following EtOH (4%) or NaCl (100 mM) addition in the wild type and a *cpdR* deletion mutant. Transcript levels were measured by quantitative RT-PCR. (e) Phase contrast images and flow cytometry data of the *cpdR* deletion mutant following stress treatment with EtOH (4%) or NaCl (100 mM).

The observed effect on the expression of CtrA-regulated genes strongly points to altered CtrA function in response to certain stress conditions. Consistently, when we monitored steady-state protein levels of CtrA by Western blotting, we found that the level of CtrA strongly and rapidly decreased upon exposure to salt, ethanol stress or mild heat shock (Fig 5A). Most conspicuously, treatment with 4% EtOH resulted in complete elimination of CtrA within only 15 minutes. Exposure to NaCl or 40°C for 60 minutes led to a drop in CtrA levels to 15% and 10%, respectively. By contrast, in response to mitomycin-induced DNA damage CtrA levels were not significantly affected. In a strain depleted of the protease subunit ClpX and in a *ΔcpdR* mutant CtrA was stabilized (Fig 5B), indicating that the ClpXP protease along with the CpdR adaptor, which are responsible for the cell cycle-dependent degradation of CtrA under optimal growth conditions [12,17], are also required to rapidly downregulate CtrA in response to environmental stress.

To test if the stress-induced reduction of CtrA steady-state levels was caused by increased proteolysis, we measured CtrA degradation rates by *in vivo* stability assays under the different stress conditions. The degradation rate of CtrA depended strongly on the external condition under which cells were cultured (Fig 5C). Under optimal growth conditions CtrA had a half-life of approximately 26 minutes, similar to previously published results [22]. Exposure to increased external salt concentrations, or incubation at 40°C led to a strong decrease in half-life to 12.8 minutes or five minutes, respectively. Most remarkably, upon incubation with 4% EtOH the half-life was as short as two minutes (Fig 5C, S4 Fig). The decrease in CtrA stability under these conditions contrasts the changes in CtrA stability induced by carbon starvation [29]; under this condition the half-life was prolonged to 173 minutes (Fig 5C). Taken together, our data demonstrate that salt, ethanol and mild heat shock induce rapid proteolysis of CtrA. More generally, our data indicate that the rate of CtrA proteolysis is subject to modulation by environmental signals allowing for the integration of external information into the cell cycle.

### Stress-induced proteolysis of CtrA is coupled to its inactivation

How do environmental stress conditions affect the rate of CtrA proteolysis? Because CtrA stability is tightly linked to its phosphorylation [15,17], we thought that the observed stress-induced decrease in CtrA stability might result from its dephosphorylation and inactivation involving the CckA-ChpT phosphorelay. To test this possibility, we measured CtrA activity following stress treatment in the *ΔcpdR* strain, in which CtrA is stabilized but growth rate unchanged under non-stress conditions (Fig 5B, S5 Fig). As a read-out for CtrA's transcription factor activity we measured the expression of CtrA-dependent genes by quantitative RT-PCR. Our data showed that as in wild type cells the mRNA abundance of the genes *sciP*, *fljO*, *ccrM*, *ftsQ* and *pilA* rapidly dropped in *ΔcpdR* cells following EtOH or NaCl addition (Fig 5D), suggesting that despite being stabilized, CtrA is not able to activate the expression of these genes after stress exposure. In agreement with our qRT-PCR data, we found that the phenotype of the *ΔcpdR* strain was indistinguishable from the filamentous phenotype of wild type cells upon treatment with EtOH or NaCl (Fig 5E, Fig 1A). These data let suggest that the increased proteolysis of CtrA under stress conditions follows its inactivation as a transcription factor.

### The rapid stress-induced inactivation of CtrA requires CckA phosphatase activity

**Fig 6:**
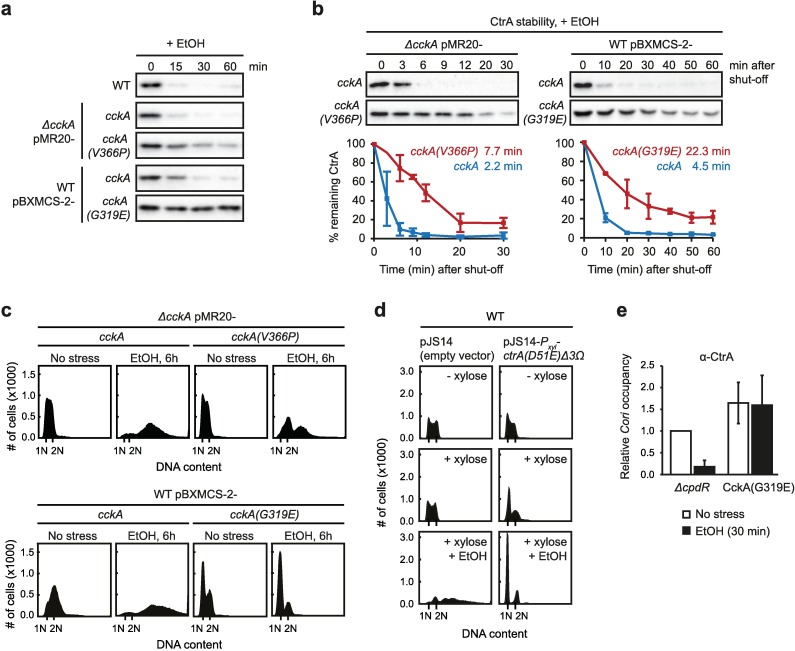
CckA phosphatase activity is required for the stress-dependent downregulation of CtrA. (a) CtrA steady-state levels during EtOH stress in the wild type and strains expressing mutant variants of CckA that either lack significant phosphatase activity (V366P) or possess increased kinase activity (G319E). *cckA(V366P)* was constitutively expressed from a low copy plasmid in a strain background lacking the native copy of *cckA*. *cckA(G319E)* was expressed from a xylose inducible promoter on pBXMCS-2 in a strain haboring wild type *cckA* at its native locus. *cckA(G319E)* expression was induced with 0.3% xylose one hour prior to ethanol addition. The data for both mutants are shown in comparison to controls, in which wild type *cckA* was expressed from the respective construct. (b) *In vivo* degradation assays of CtrA during EtOH (4%) treatment in cells expressing *cckA(V366P)* or *cckA(G319E)* in comparison to cells expressing wild type *cckA*. The expression of *cckA* and *cckA(G319E*) was induced with 0.3% xylose one hour prior to ethanol addition. Cells were incubated for five minutes with 4% EtOH before chloramphenicol was added to shut-off protein synthesis. Intensities of the Western blot bands were quantified and averages of two independent replicates with standard deviations are shown. CtrA half-lives are indicated. See also S6 Fig for stability assays under NaCl stress. (c) Flow cytometry profiles of the *cckA(V366P)* and *cckA(G319E)* mutants without stress and after 6 hours EtOH (4%) treatment. The expression of *cckA* and *cckA(G319E)* on pBXMCS-2 was induced with 0.3% xylose one hour prior to EtOH addition. See also S7 Fig for other stress conditions. (d) Flow cytometry profiles of cells expressing *ctrA(D51E)Δ3Ω* with and without EtOH (4%, 6h) treatment in comparison to the empty vector control. Cultures were incubated for 30 minutes with xylose and then treated with NaCl (100 mM) or ethanol (4%). See also S8 Fig for NaCl stress. (e) qChIP data showing CtrA occupancy of *Cori* (*c* and *d* CtrA binding sites) in the *ΔcpdR* mutant and *cckA(G319E)*-expressing cells in the absence of stress and after 30 minutes treatment with 4% ethanol. Expression of pBXMCS-2-P_*xyl*_*-cckA(G319E)* was induced with xylose for one hour prior to stress treatment. Each data point represents the average of two independent experiments with standard deviations.

Under non-stress conditions CckA dynamically switches between its kinase and phosphatase activities [16,34]. We reasoned that the observed inactivation of CtrA could either be due to complete loss of CckA function or to a more active mechanism, in which CckA switches from a kinase into a phosphatase. To distinguish between these possibilities we investigated if CckA phosphatase activity is required to induce rapid downregulation of CtrA upon EtOH treatment, the stress condition that impacted CtrA stability most strongly. To this end, we used a point mutant of CckA, CckA(V366P), that retains kinase activity, but lacks significant phosphatase activity [16]. Indeed, constitutive expression of this mutant in a *cckA* deletion background partially prevented CtrA downregulation upon EtOH addition, whereas expression of wild type CckA from the same construct led to rapid removal of CtrA similar to the wild type (Fig 6A). Monitoring CtrA degradation rates in *cckA(V366P)*-expressing cells showed that the half-life of CtrA during EtOH exposure was markedly increased in the mutant (7.7 min) compared to the wild type (2.2 min) (Fig 6B). Similar results were also obtained under NaCl stress (S6 Fig). Together, these data indicate that CckA phosphatase activity is critical to ensure rapid degradation of CtrA in response to external stress.

We next tested if increasing the kinase activity of CckA results in a similar effect by using a CckA variant containing the substitution G319E, which has been shown to render CckA hyperactive as a kinase [16]. Because constitutive expression of *cckA(G319E)* leads to severe cell cycle and growth defects, we expressed this variant from an inducible promoter in a wild type background. Western blots showed that CtrA levels were completely stabilized in this mutant following EtOH treatment (Fig 6A). Moreover, the half-life of CtrA in cells expressing this mutant was strongly increased in the presence of stress (22.3 min) compared to the half-life in cells expressing wild type CckA (4.5 min) (Fig 6B, S6 Fig). Consistent with the strong stabilization of CtrA in *cckA(G319E)*-expressing cells we observed that cells neither formed filaments nor accumulated chromosomes when treated with salt, EtOH or mild heat shock, but instead arrested with a single chromosome in G1-phase (Fig 6C, S7 Fig). Expression of a stable and active *ctrA* allele, *ctrA(D51E)Δ3Ω* [13] led to a similar phenotype: cells no longer accumulated multiple chromosomes in response to EtOH and NaCl stress and instead arrested in G1-phase (Fig 6D, S8 Fig). These data suggest that the replication phenotype of *cckA(G319E)* cells is due to increased CtrA phosphorylation and activity. We observed that stress-induced cell filamentation and chromosome accumulation was also less severe in *cckA(V366P)* cells than in the wild type (Fig 6C, S7 Fig). However, compared to the *cckA(G319E)* and the *ctrA(D51E)Δ3Ω* mutants, the change of phenotype was not as strong, likely because CtrA was only partially stabilized in the *cckA(V366P)* mutant (Fig 6A).

Together our data are consistent with a model, in which environmental stress causes CckA to switch to phosphatase activity, leading to CtrA dephosphorylation and rapid proteolysis. As an additional verification of this model, we analyzed CtrA activity by measuring its occupancy at the origin of replication (*Cori*) upon stress treatment by using quantitative chromatin immunoprecipitation (qChIP). We compared CtrA occupancy between the *ΔcpdR* mutant and the *cckA(G319E)* mutant, in both of which CtrA is completely stbilized upon stress exposure (Fig 5B, Fig 6A). In the *ΔcpdR* mutant addition of EtOH led to a decrease in CtrA occupancy to 18% (Fig 6E), indicating that stress treatment results in CtrA dephosphorylation and inactivation. By contrast, in cells expressing *cckA(G319E)*, CtrA occupancy at *Cori* remained high even after stress exposure (Fig 6E). These results reinforce our conclusion that stress triggers the inactivation and proteolysis of CtrA by stimulating CckA phosphatase activity.

### Stress-induced CtrA inactivation and degradation does not depend on DivK-DivL and c-di-GMP

How is CckA phosphatase activity stimulated under stress conditions? Previous work has reported different mechanisms that allow CckA to switch between its kinase and its phosphatase mode under standard conditions [22,24]. One such mechanism depends on the upstream regulatory factors DivL and DivK. While DivL directly interacts with CckA at the swarmer pole in stalked and predivisional cells and activates its kinase activity [23], DivK acts as an antagonist of DivL-CckA favoring the phosphatase mode of CckA at the stalked pole [22].

**Fig 7:**
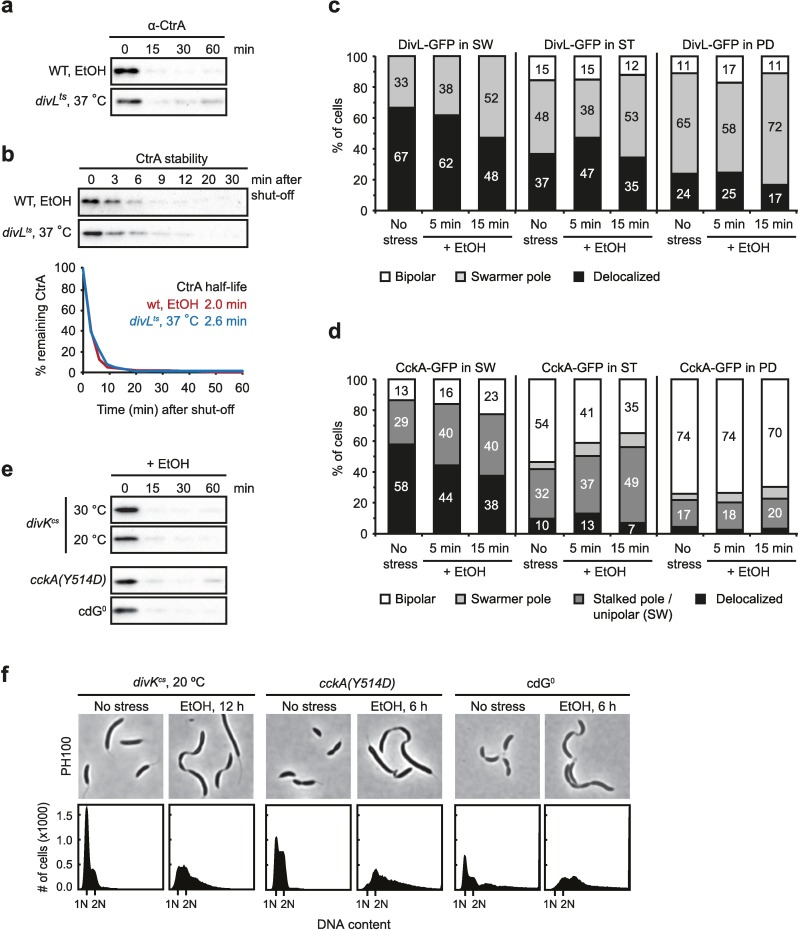
The stress-induced CtrA inactivation and degradation depends neither on DivK-DivL nor on c-di-GMP. (a) Steady-state levels of CtrA in *divL*^*ts*^ cells after shift to the restrictive temperature (37°C) in comparison to CtrA levels in wild type cells treated with EtOH. (b) CtrA stability in the *divL*^*ts*^ mutant after shift to the restrictive temperature in comparison to CtrA stability following EtOH addition in wild type cells. Stress induction or temperature shift was performed five minutes prior to chloramphenicol addition. The bottom graphs show quantifications of band intensities; CtrA half-lives are indicated. (c) Quantification of fluorescence microscopy data analyzing the subcellular localization of DivL-GFP in swarmer cells (SW), stalked cells (ST), and predivisional cells (PD) in the absence of stress (no stress) and after five and 15 minutes treatment with 4% EtOH. Percentage of cells displaying either bipolar DivL-GFP localization, foci at the swarmer pole or delocalized DivL-GFP are indicated within the bars. See also S9 Fig for representative images and illustrations. (d) Quantification of microscopy data analyzing the localization pattern of CckA-GFP (bipolar, swarmer pole, stalked pole / unipolar (SW), and delocalized) in the different cell types during EtOH stress. (e) CtrA steady-state levels following EtOH (4%) addition in a *divK*^*cs*^ mutant under permissive (30°C) and restrictive (20°C) conditions, in a *cckA(Y514D)* mutant and in a cdG^0^ strain. To ensure inactivation of DivK, *divK*^*cs*^ cells were cultured for three hours at 20°C prior to EtOH treatment. (f) Cell morphology and chromosome content in *divK*^*cs*^ cells (when grown under the restrictive temperature 20°C), the *cckA(Y514D)* mutant and the cdG^0^ strain following treatment with 4% EtOH. *divK*^*cs*^ cells were stress-treated for twelve hours (instead of six hours) due to the slower growth rate at low temperature.

Consistent with DivL's role in activating CckA kinase activity, previous work showed that loss of DivL function leads to a strong reduction in the level of phosphorylated CtrA and rapid CtrA degradation [22]. In agreement with these data we found that shifting a *divL*^*ts*^ mutant strain to the restrictive temperature resulted in rapid proteolysis and downregulation of CtrA (Fig 7A and 7B) [22]. Because the rate of CtrA degradation in the *divL*^*ts*^ strain was similarly fast as in wild type cells upon EtOH treatment, we hypothesized that stress-dependent inactivation and proteolysis of CtrA might be caused by a failure to accumulate and localize DivL. However, our microscopy data showed that fluorescently tagged DivL (DivL-GFP) correctly localized in a cell cycle-dependent manner even in the presence of EtOH (Fig 7C, S9 Fig). Similarly, we did not observe that EtOH treatment induced significant changes in the localization pattern of CckA that changes during the cell cycle (Fig 7D, S9 Fig) [35]. By contrast, loss of DivL function has previously been shown to cause mislocalization of CckA [22,23]. Together, these data suggest that CtrA inactivation in response to adverse conditions is not due to failure to accumulate or localize DivL.

To investigate whether the switch of CckA into its phosphatase mode is caused by increased activity of DivK, we measured CtrA levels following EtOH treatment in a strain in which DivK was inactivated. When shifting a cold-temperature sensitive mutant of DivK (*divK*^*cs*^) to the restrictive temperature 20°C [22,36], we observed the same rapid downregulation of CtrA upon EtOH treatment as in the wild type (Fig 7E), suggesting that the stress-dependent regulation of CtrA activity is independent of DivK.

In addition to the upstream regulators DivK and DivL, it has recently been shown that c-di-GMP can modulate CckA activity through a direct interaction. Binding of c-di-GMP to CckA inhibits the kinase and stimulates the phosphatase activity of CckA [24]. To test the possibility that EtOH affects CckA activity through c-di-GMP, we investigated if the EtOH-dependent changes in CtrA abundance and stability were abolished in a CckA(Y514D) mutant that is compromised for c-di-GMP binding [24]. However, just like in the wild type, addition of EtOH resulted in rapid CtrA downregulation in this mutant (Fig 7E). Similarly, we found that in a strain lacking all diguanylate cyclases (cdG^0^) that is devoid of c-di-GMP [25], EtOH addition resulted in the same drop in CtrA abundance as in the wild type (Fig 7E). Consistent with the downregulation of CtrA in *divK*^*cs*^, CckA(Y514D) and cdG^0^ cells, we observed that EtOH-treatment led to the similar phenotypic changes as observed in wild type cells; cells became filamentous and accumulated multiple chromosomes (Fig 7F, Fig 1A).

Altogether these data suggest that external EtOH stress affects CckA and CtrA activity neither via the DivK-DivL pathway nor through c-di-GMP signaling. Hence, the histidine kinase might directly respond to external changes.

### Efficient downregulation of CtrA is required to maintain growth and cell integrity

**Fig 8:**
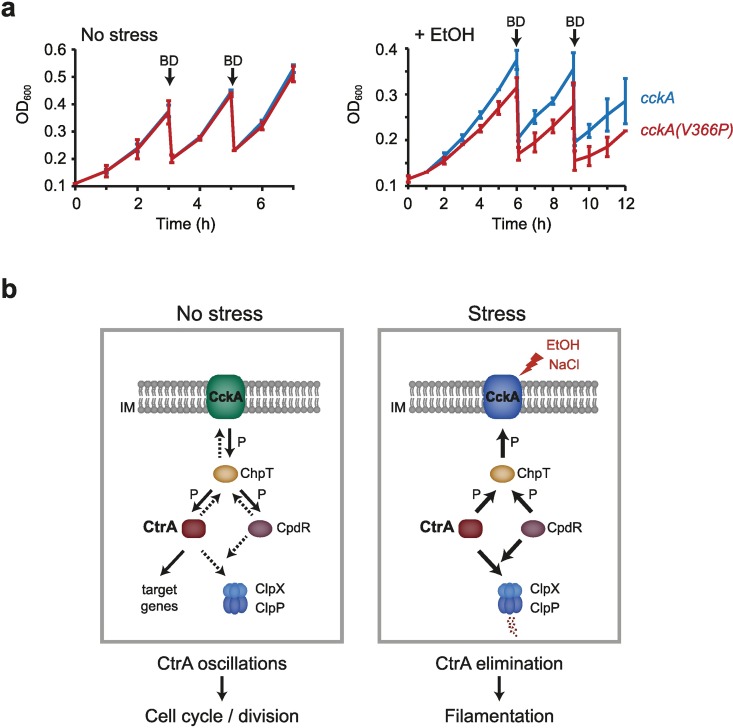
Efficient downregulation of CtrA is required to maintain growth and cell integrity. (a) Growth curves of cells expressing wild type *cckA* or *cckA(V366P)* in the absence of stress or in the presence of 4% EtOH. The averages of two independent replicates are shown with standard deviations. Cultures were back-diluted (BD) at the indicated time points to monitor growth over a longer period of time without reaching stationary phase. (b) Proposed model of the stress-dependent regulation of CtrA and cell division through the CckA-ChpT phosphorelay. Left panel shows the CckA phosphorelay under optimal conditions, CckA switching between its kinase and phosphatase mode permits cell cycle-dependent oscillations of CtrA, cell division and cell cycle progression. By contrast, stress conditions such as EtOH or NaCl lock CckA in its phosphatase mode, resulting in CtrA dephosphorylation and elimination and consequently a block of cell division.

We wondered whether the stress-dependent downregulation of CtrA provides a selective advantage and helps the culture to survive and grow under adverse conditions. To investigate the physiological relevance of the rapid shut-down of CtrA function during ethanol stress, we compared the growth rate of wild type cells with that of *cckA(V366P)* mutant cells, in which CtrA is partially stabilized due to lower CckA phosphatase activity (Fig 6A and 6B). Under optimal conditions, the wild type and the mutant had identical growth rates suggesting that CckA phosphatase activity is not critical for cell growth and cell cycle progression in the absence of stress (Fig 8A). Interestingly however, in the presence of 4% EtOH the *cckA(V366P)* mutant strain showed a clearly reduced growth rate compared to the wild type. This result suggests that under stress conditions the shut-down of CtrA function due to increased CckA phosphatase activity indeed provides a growth advantage.

## Discussion

This work reports a new mechanism by which bacteria delay cell division and consequently transform into filamentous cells under stress. Unlike previously described mechanisms that transiently block cell division through the induction of small division inhibitors [26,27], the mechanism that we describe depends on a phospho-signaling system that modulates the stability and activity of a master cell cycle regulator required for cell division.

The bifunctional histidine kinase CckA plays a central role in this regulation as it makes the decision of whether to divide or not by integrating the cell cycle with environmental information. It is well established that under optimal conditions CckA drives oscillations of the master cell cycle regulator CtrA through dynamically switching between its kinase and phosphatase activities [16,22,34,37]. Our new data suggest that environmental stress locks CckA in its phosphatase mode leading to the rapid inactivation of CtrA, its elimination through the protease ClpXP and consequently a block of CtrA regulated functions including cell division (Fig 8B). Although our data show that CckA phosphatase activity is critical for the stress-dependent inactivation of CtrA, the detailed molecular process by which stress signals modulate CckA function remains to be elucidated. Our results rule out the involvement of the small signaling molecule c-di-GMP (Fig 7E and 7F), which promotes CckA phosphatase activity at the G1-to-S transition and promotes CtrA degradation by ClpXP under non-stress conditions [21,24,37]. Similarly, the stress-dependent regulation does not appear to be mediated by the upstream regulators DivL and DivK (Fig 7), which are critical for the cell cycle-dependent regulation of CckA in the absence of stress [22].

The result that none of the known CckA regulators were involved in the stress-dependent regulation of CckA together with the rapid response time that we observed (Fig 5) argues for a direct sensing mechanism. CckA has two PAS (Per Arnt Sim) domains, PAS-A and PAS-B, which sense distinct spatiotemporal signals and thereby mediate the cell cycle-dependent regulation of CckA activity [34]. These PAS domains might also perceive information about the environment, for example by binding molecules that are present under certain conditions. Alternatively, stress-sensing could be mediated in a fashion independent of CckA's PAS domains and instead involve its periplasmic or transmembrane regions. Indeed, previous work on other kinases demonstrated that environmental information can be directly sensed through the membrane [38]. For example, the histidine kinase DesK from *Bacillus subtilis* was shown to respond to temperature changes by sensing membrane thickness [39]. It is well documented that salt, EtOH and increased temperature induce changes in membrane properties, for example membrane fluidity or lipid composition [40–42]. These changes might directly induce changes in CckA conformation and activity. A direct sensing mechanism by CckA would provide an efficient means to transduce environmental information into the cell cycle. Nevertheless, we do not rule out the involvement of unidentified regulatory proteins that may interact with CckA to control its activity in response to stress.

Other studies have analyzed the response of *C*. *crescentus* to increased salt concentrations. One of them investigated gene expression and proteome changes upon treatment with 60 mM NaCl [43]. Under this condition growth rate and CtrA regulated genes were hardly affected [43], which is consistent with our finding that the salt-induced filamentous phenotype occurred only in a narrow range of concentrations (Fig 1B). Another recent study investigated the response of individual cells of *C*. *crescentus* to repeated salt exposure using a microfluidics system [44]. The authors observed that an initial exposure to moderate NaCl concentrations (80 mM) led to a cell division delay and cell-cycle synchronization and that the response of individual cells to a subsequent exposure to a higher NaCl concentration (100 mM) was dependent on the cell cycle state [44]. It is possible that the stress-induced changes in CtrA activity that we report here contribute to these behaviors.

Noticeably, while salt stress, EtOH stress and mild heat shock lead to rapid elimination of CtrA, our data as well as previously published results show that carbon starvation causes an increase in CtrA stability by a mechanism involving the small signaling molecule (p)ppGpp [28,29,45]. Although the precise mechanism of the starvation-dependent increase in CtrA stability remains unclear, it likely ensures, in combination with the downregulation of the DNA replication initiator DnaA, a block of DNA replication initiation under this condition [1,28,29].

Besides elucidating the mechanisms of *how* cell division is environmentally controlled, another important question concerns *why* cells inhibit cell division under stress. Cell division is a vulnerable process involving extensive membrane and cell wall remodeling [46]. Initiating this process under stress conditions, in particular those impacting the cell membrane, potentially causes cell lysis and consequently death. Preventing cell division in the presence of stress might thus provide a mechanism to preserve cell integrity. Continued global macromolecule synthesis, which still can take place under the conditions that we described (Fig 1A, S2 Fig), allows for the production of new cell mass, enabling the rapid generation of new daughter cells when conditions improve. It is also possible that the filamentous morphology of division-inhibited cells provides an adaptive advantage under certain conditions in nature. A filamentous cell shape is expected to influence various cell properties, including surface area, mobility and adhesive forces, and is thus expected to affect the interaction with other species and the attachment of cells to biotic and abiotic surfaces [3,4].

Finally, while we have focussed in this study on *C*. *crescentus*, other alphaproteobacteria might employ similar mechanisms to control CtrA and cell division in response to external cues. Previous work in the nitrogen-fixing plant symbiont *Sinorhizobium meliloti* demonstrated that CtrA is strongly downregulated during the early steps of symbiosis [47]. In the pathogen *Brucella abortus* the CckA-ChpT-CtrA-CpdR pathway was shown to be required for intracellular survival in human macrophages [48]. Therefore, precise environmental regulation of CtrA abundance and activity likely plays an important role for the diverse functions that different alphaproteobacteria perform in the environment.

## Materials and Methods

### Growth conditions

Wild type *C*. *crescentus* NA1000 and its mutant derivatives were routinely grown in PYE (rich medium) or M2G medium (minimal medium containing 0.2% glucose). When necessary, growth medium was supplemented with 0.3% xylose, 0.2% glucose or 1 mM IPTG. Cultures were grown at 30°C with 200 rpm, temperature sensitive mutants were cultivated at 30°C and sensitivity was induced either at 37°C or 20°C depending on the mutant allele. Antibiotics were added as previously described [30,49]. Rifampicin was used at concentrations of 2.5 μg/ml (liquid media) and 5 μg/ml (solid media) for *C*. *crescentus* and 25 μg/ml (liquid media) and 50 μg/ml (solid media) for *E*. *coli*. *E*. *coli* strains were routinely grown in LB medium at 37°C, supplemented with antibiotics as required.

For induction of stress, mixed (non-synchronized) *Caulobacter* cultures grown overnight to exponential phase in PYE medium (OD 0.1–0.4) were shifted to medium supplemented with NaCl, EtOH, mitomycin C, sucrose or H_2_O_2_ at the indicated concentrations. To induce heat shock, cultures grown at 30°C were diluted in pre-heated medium and cultivated at 37, 39, 40, 42 or 45°C for the indicated time. Carbon starvation was induced by shifting cells from M2G to M2 medium supplemented with 0.02% glucose as previously described [28]. Low and high pH stress medium was prepared by adjusting the pH with HCl or Na_2_CO_3_ and NaHCO_3_ [32] to the pH values of 4.9 and 9.1, respectively. If necessary, cultures were backdiluted during the stress treatment to keep them in exponential phase.

### Strain construction

Strains used in this study are listed in S1 Table. Strain Δ*cpdR*::*rif*, KJ798, was created by using the two-step recombination procedure [50] with plasmid pKJ808. Strain KJ799 was generated by introducing the plasmid pKJ809 into *C*. *crescentus* NA1000 by electroporation. To construct strain KJ800, plasmid pKJ810 was introduced instead. To construct strain KJ811 the empty vector pJS14 was introduced into *C*. *crescentus* NA1000 by electroporation.

### Plasmid construction

#### pKJ808

600 bp upstream (flank1) and 600 bp downstream (flank2) of the *cpdR* coding sequence were amplified by PCR with the primer pairs OKH55/59 and OKH58/60, respectively. The *rif* cassette was amplified using primers OKH53 and OKH64. Plasmid pNPTS138 was amplified using primers OFS285 and OFS286. All fragments were assembled by Gibson assembly [51] resulting in plasmid pKJ808.

#### pKJ809

For amplifying the *cckA* fragment, PCR was performed with the primers OKH71 and OKH72 using a strain harboring *cckA* on a plasmid as template. Plasmid pBXMCS-2 was amplified using primers OFS321 and OFS360. Fragments were assembled by Gibson assembly resulting in pKJ809.

#### pKJ810

The construction of pKJ810 followed the same procedure as pKJ809. The *cckA(G319E)* fragment was amplified with the same primers on a template that contained the mutated version of *cckA* [16].

### Flow cytometry

Samples were prepared as earlier described [28] and analyzed using a BD LSRFortessa flow cytometer or the BD LSR II (BD Biosciences). Data were collected for at least 30000 cells. Flow cytometry data were analyzed with FlowJo. Each experiment was repeated independently and representative results are shown.

### Microscopy

For living cell analysis and time-lapse microscopy, cells were transferred onto a PYE 1% agarose pad with supplementation as required. Otherwise cells were fixed with 1% formaldehyde, pelleted, resuspended in an appropriate volume of ddH_2_O and mounted onto 1% agarose pads. Phase contrast and fluorescence images were taken using a T*i* eclipse inverted research microscope (Nikon) with a 100x/1.45 NA objective (Nikon). Fiji (ImageJ) was used for image processing.

### Plate reader experiment

One ml culture was harvested and resuspended in 1 ml ddH_2_O. The optical density and the GFP fluorescence of 100 μl cells was measured in a SpectraMax i3x (Molecular Devices) plate reader. The fluorescence / OD ratio was calculated after blanking and the WT auto fluorescence signal was subtracted from the GFP signal.

### Immunoblotting

Pelleted cells were resuspended in 1X SDS sample buffer, normalized to the optical density of the culture and heated to 95°C for 10 min. Protein extracts of cell lysates were then subject to SDS-PAGE for 90 min at 130 V at room temperature on 11% Tris-glycine gels and transferred to nitrocellulose membranes. To verify equal loading, total protein was visualized using the TCE in-gel method [52] prior to blotting. Proteins were detected using primary antibodies against CtrA (kindly provided by M. Laub), DnaK or FtsZ (kindly provided by M. Thanbichler) in appropriate dilutions, and a 1:5000 dilution of secondary HRP-conjugated antibody. SuperSignal Femto West (Thermo Scientific) was used as detection reagent. Blots were scanned with a Chemidoc (Bio-Rad) system. Images were processed with Bio-Rad Image Lab, Adobe Photoshop, Image J and the relative band intensities quantified with Image Lab software.

### *In vivo* degradation assay

To measure protein degradation *in vivo*, cells were grown under the desired conditions. Protein synthesis was blocked by addition of 100 μg/ml chloramphenicol. Samples were taken as indicated, every 10 min (for 1 hour) or at the time points 0, 3, 6, 9, 12, 20, and 30 minutes, and pellets were snap frozen in liquid nitrogen before being analyzed by Western blotting.

### RNA sequencing

RNA was collected from bacteria that were grown under the appropriate conditions and extracted using the RNeasy mini kit (Qiagen). RNA-sequencing was performed by GENEWIZ, South Plainfield, NJ. For statistical analysis, the transcriptome data of the EtOH and NaCl stress conditions were compared to each other, to *divL*^*ts*^ transcriptome data and to previously published DNA damage microarray [26]. In order to integrate RNA-seq and microarray transcriptomics data we applied a fold-change cut-off of two to identify up- and down-regulated genes. For each condition, genes with a fold-change > 2 in the stress / non-stress sample comparison were assigned to the up-regulated group and genes with a fold-change < 0.5 to the down-regulated group. The similarity between two conditions is reflected by the extent of intersection of the genes in the respective up-regulated and down-regulated gene groups. As the intersection depends strongly on the size of the groups we used Monte-Carlo simulations to statistically evaluate group intersections. The z-score indicates the number of standard deviations from a random intersection. Hence, a high z-score means a strong deviation from a random similarity value.

### qChIP

Samples were prepared as previously described [53] with the following modification: Lysates from one independent experiment were sonicated simultaneously (Bioruptor *Plus*, diagenode, Ougrée, BE) using 40 cycles of 30 seconds sonication (High) and 30 seconds pause. Real-time PCR was performed with a StepOnePlus real-time PCR system (AppliedBiosystems, Foster City, CA) using 5% of each ChIP sample and the SYBR green PCR master mix (Bio-Rad) in a 20 μl volume and 10 pmol primers (OKH79 and OKH80), amplifying a 88 bp region spanning the *c* and *d* CtrA binding boxes within *Cori*. The cycle threshold (Ct) of the input DNA was adjusted to 100%. The percentage of the input DNA was calculated (100*2^(adjusted input DNA-Ct(IP))) for every condition and mutant. Each qPCR reaction was performed in triplicate.

### Quantitative RT-PCR

RNA was collected from bacteria that were grown under the appropriate conditions as described above. Equal amounts of isolated RNA were reverse transcribed into cDNA using the iScript cDNA synthesis kit (Bio-rad). The cDNA was used as template for the real-time PCR reaction using the iTaq universal SYBR Green Supermix (Bio-rad) and primers as listed in S2 Table. Analysis was performed with a qTower instrument (Analytik Jena) using the standard run mode. For detection of primer dimerization or other artifacts of amplification, a dissociation curve was run immediately after completion of the real-time PCR. Individual gene expression profiles were normalized against 16S RNA, serving as an endogenous control. Relative expression levels were determined with the comparative Ct method. Each qPCR reaction was performed in triplicate.

### Accession numbers

Complete data sets for the RNA-seq experiment are provided in S3 Table in the supplemental material and are available through GEO (accession number GSE90030).

## Supporting Information

S1 Fig Subcellular localization of origins of replication in cells treated with 100 mM NaCl, 4% ethanol or 40°C. Origins were visualized using a FROS-reporter in which TetR-YFP binds to a *tetO* array integrated close to the origin. Expression of *tetR-yfp* was induced with 0.3% xylose two hours prior to imaging. Cells were treated for six hours with NaCl, EtOH, or 40°C heat shock before being analyzed.
(EPS)

S2 Fig Growth rate and viability of wild type *Caulobacter* cells treated with different NaCl or EtOH concentrations, or at different temperatures. (a) Growth curves of wild type cultures when treated with different concentrations NaCl or EtOH or when cultivated at different temperatures. Growth was monitored by measuring the increase in optical density over time. (b) Viability assays under different stress conditions. Wild type cultures were treated with the indicated stress condition for the indicated time. Dilutions of the stress-treated cultures were then spotted on PYE plates without supplements and incubated for two days at 30°C.
(EPS)

S3 Fig Quantification of *P*_*sidA*_*-egfp* expression using a plate reader. Cultures were treated for two hours with 3 μg/ml mitomycin C (MMC, positive control), 100 mM NaCl, 4% EtOH or 40°C.
(EPS)

S4 Fig *In vivo* degradation assays of CtrA upon EtOH (4%) addition or shift to 40°C. Wild type cells were stress-treated for five minutes before chloramphenicol was added to shut-off protein synthesis. CtrA abundance was monitored by Western blotting. Intensities of the bands were quantified and averages of two independent replicates with standard deviations are shown in Fig 5C.
(EPS)

S5 Fig Growth curve of a Δ*cpdR* deletion mutant in comparison to the wild type under non-stress conditions. (EPS)

S6 Fig *In vivo* degradation assays of CtrA during NaCl (100 mM) treatment in cells expressing *cckA(V366P)* or *cckA(G319E)* in comparison to cells expressing wild type *cckA*. The expression of *cckA* and *cckA(G319E*) was induced with 0.3% xylose 1 hour prior to NaCl addition. Cells were incubated for five minutes with NaCl before chloramphenicol was added to shut-off protein synthesis. Intensities of the Western blot bands were quantified; averages of two independent replicates with standard deviations are shown. CtrA half-lives are indicated.
(EPS)

S7 Fig DNA content and cell morphology of *cckA(V366P)* and *cckA(G319E)* expressing cells when treated with 100 mM NaCl, 4% EtOH or 40C heat shock. (a) Phase contrast microscopy images and flow cytometry profiles of cells expressing *cckA(V366P)* or wild type *cckA* (as a control) after treatment with 100 mM NaCl, 4% EtOH or 40°C. (b) Phase contrast images and flow cytometry profiles of *cckA(G319E)* expressing cells or wild type *cckA* (as a control) after treatment with 100 mM NaCl, 4% EtOH or 40°C.
(EPS)

S8 Fig Flow cytometry profiles of cells expressing *ctrA(D51E)Δ3Ω* with and without NaCl treatment. Cultures were incubated for 30 minutes with xylose and then treated with NaCl (100 mM, 6h).
(EPS)

S9 Fig Representative images showing the subcellular localization of DivL-GFP and CckA-GFP upon EtOH treatment. The localization of DivL-GFP (upper panel) and CckA-GFP was assessed by fluorescence microscopy of swarmer cells (SW), stalked cells (ST) and pre-divisional cells before and after five and 15 minutes EtOH (4%) stress. The percentage of cells displaying a certain localization pattern (delocalized or swarmer pole localization for DivL-GFP; delocalized, unipolar, stalked pole or bipolar for CckA-GFP) is indicated. A schematic illustration for each localization pattern is shown.
(EPS)

S1 Table Strains and plasmids used in this study. (DOCX)

S2 Table Sequences of the primers used in this study. (DOCX)

S3 Table RNA-sequencing data. (XLSX)
