# Supplementary material for: A Kinase-Phosphatase Switch Transduces Environmental Information into a Bacterial Cell Cycle Circuit
Source: PLoS Genet. 2016 Dec 12;12(12):e1006522. doi: 10.1371/journal.pgen.1006522 (PMC5189948; doi:10.1371/journal.pgen.1006522)
Supplement: S1 Table — (DOCX) [file pgen.1006522.s010.docx]

| **Strains** | **Description** | **Reference** |
| --- | --- | --- |
| ***C. crescentus*** |  |  |
| NA1000 | Synchronizable derivative of wild-type CB15 | [[1](#_ENREF_1)] |
| KJ798 | *ΔcpdR::rif* | This study |
| KJ799 | NA1000 + pBXMCS-2-*P_xyl_-cckA* | This study |
| KJ800 | NA1000 + pBXMCS-2-*P_xyl_-cckA(G319E)* | This study |
| KJ811 | NA1000 + pJS14 | This study |
| LS3313 | *divK341^cs^* | [[2](#_ENREF_2)] |
| ML1491 | Δ*cckA::*gent + pMR20-P*_cckA_*-*cckA* | [[3](#_ENREF_3)] |
| ML1497 | Δ*cckA::*gent + pMR20-P*_cckA_*-*cckA(V366P)* | [[3](#_ENREF_3)] |
| ML1509 | NA1000 + pJS14-*P_xylX_::ctrA(D51E)Δ3Ω* | Laub lab |
| ML1681 | *P_cckA_*-*cckA-gfp* | [[3](#_ENREF_3)] |
| ML1756 | *divL-egfp::gent* | [[4](#_ENREF_4)] |
| ML1852 | *divL^ts^* | [[2](#_ENREF_2)] |
| ML1890 | NA1000 + pCT133-P*_sidA_-egfp* | [[5](#_ENREF_5)] |
| ML2032 | Δ*clpX* Δ*socB* + pMR20:P*_lacI_‐lacI*‐P*_lac_*-c*lpX::tet^R^* | [[6](#_ENREF_6)] |
| MT16 | *ori::(tetO)_n_* *xylX*::pHPV472 (LacI-CFP and TetR-YFP) | [[7](#_ENREF_7)] |
| MT196 | *P_vanA_-ftsZ-eyfp* | [[8](#_ENREF_8)] |
| UJ5065 | cdG^0^ NA1000 *∆dgcB ∆cc0857 ∆cc0740 ∆dgcA ∆pleD ∆cc0655 ∆cc3094 ∆cc0896* | [[9](#_ENREF_9)] |
| UJ7212 | *cckA(Y514D)* | [[10](#_ENREF_10)] |
| ***E. coli*** |  |  |
| DH5α | General cloning strain | Invitrogen |
| **Plasmids** |  |  |
| pNPTS138 | Integration vector | Lab collection |
| pBXMCS-2 | Medium-copy vector for xylose-inducible gene expression | [[11](#_ENREF_11)] |
| pCT133 | Destination vector of pMR20, low copy plasmid | Lab collection |
| pJS14 | Medium-copy expression vector | Lab collection |
| pXylX::ctrAD51EΔ3Ω | pJS14-*P_xylX_::ctrA(D51E)Δ3Ω* | [[12](#_ENREF_12)] |
| pKJ808 | pNPTS138; flanks for *cpdR* deletion | This study |
| pKJ809 | pBXMCS-2 P*_xyl_-cckA* | This study |
| pKJ810 | pBXMCS-2 P*_xyl_-cckA(G319E)* | This study |

**S1 Table. Strains and plasmids used in this study.**

**References**

1. Evinger M, Agabian N (1977) Envelope-associated nucleoid from Caulobacter crescentus stalked and swarmer cells. J Bacteriol 132: 294-301.

2. Tsokos CG, Perchuk BS, Laub MT (2011) A dynamic complex of signaling proteins uses polar localization to regulate cell-fate asymmetry in Caulobacter crescentus. Dev Cell 20: 329-341.

3. Chen YE, Tsokos CG, Biondi EG, Perchuk BS, Laub MT (2009) Dynamics of two Phosphorelays controlling cell cycle progression in Caulobacter crescentus. J Bacteriol 191: 7417-7429.

4. Chen YE, Tropini C, Jonas K, Tsokos CG, Huang KC, et al. (2011) Spatial gradient of protein phosphorylation underlies replicative asymmetry in a bacterium. Proc Natl Acad Sci U S A 108: 1052-1057.

5. Modell JW, Hopkins AC, Laub MT (2011) A DNA damage checkpoint in Caulobacter crescentus inhibits cell division through a direct interaction with FtsW. Genes Dev 25: 1328-1343.

6. Aakre CD, Phung TN, Huang D, Laub MT (2013) A bacterial toxin inhibits DNA replication elongation through a direct interaction with the beta sliding clamp. Mol Cell 52: 617-628.

7. Viollier PH, Thanbichler M, McGrath PT, West L, Meewan M, et al. (2004) Rapid and sequential movement of individual chromosomal loci to specific subcellular locations during bacterial DNA replication. Proc Natl Acad Sci U S A 101: 9257-9262.

8. Thanbichler M, Shapiro L (2006) MipZ, a spatial regulator coordinating chromosome segregation with cell division in Caulobacter. Cell 126: 147-162.

9. Abel S, Bucher T, Nicollier M, Hug I, Kaever V, et al. (2013) Bi-modal distribution of the second messenger c-di-GMP controls cell fate and asymmetry during the caulobacter cell cycle. PLoS Genet 9: e1003744.

10. Lori C, Ozaki S, Steiner S, Bohm R, Abel S, et al. (2015) Cyclic di-GMP acts as a cell cycle oscillator to drive chromosome replication. Nature 523: 236-239.

11. Thanbichler M, Iniesta AA, Shapiro L (2007) A comprehensive set of plasmids for vanillate- and xylose-inducible gene expression in Caulobacter crescentus. Nucleic Acids Res 35: e137.

12. Domian IJ, Quon KC, Shapiro L (1997) Cell type-specific phosphorylation and proteolysis of a transcriptional regulator controls the G1-to-S transition in a bacterial cell cycle. Cell 90: 415-424.
