# Supplementary material for: A Kinase-Phosphatase Switch Transduces Environmental Information into a Bacterial Cell Cycle Circuit
Source: PLoS Genet. 2016 Dec 12;12(12):e1006522. doi: 10.1371/journal.pgen.1006522 (PMC5189948; doi:10.1371/journal.pgen.1006522)
Supplement: S2 Table — (DOCX) [file pgen.1006522.s011.docx]

| **Name** | **description** | **Sequence (5' to 3')** |
| --- | --- | --- |
| OKH9 | qPCR primer for *ftsQ*, fw | CTCACAGCCACCATTTC |
| OKH10 | qPCR primer for *ftsQ*, rv | ATCAGCTCGACCAAAGG |
| OKH11 | qPCR primer for *ccrM*, fw | CCCAGCTGCAGATTATAGG |
| OKH12 | qPCR primer for *ccrM*, rv | GCATCGAGCAGATGAAC |
| OKH33 | qPCR primer for *fljO*, fw | CGCTTCCTGAAGGATGAATC |
| OKH34 | qPCR primer for *fljO*, rv | CTTCGTGAAGGCGGTGC |
| OKH35 | qPCR primer for *pilA*, fw | GGCGCTAAACAGCATCA |
| OKH36 | qPCR primer for *pilA*, rv | GTTGCTGGACGTTGTTCA |
| OKH41 | qPCR primer for *sigT*, fw | CAGGACGCGATGATGAAGG |
| OKH42 | qPCR primer for *sigT*, rv | CCGCTTTTCCGAATAGAACTG |
| OKH49 | qPCR primer for *sciP*, fw | CCGGGGTGAAAAGTACGTCA |
| OKH50 | qPCR primer for *sciP*, rv | TCAGCTTGTAACGATCGCAC |
| OKH53 | amplifiy *rif* cassette, fw | TCGAACCCCAGAGTCCCGGC |
| OKH55 | overlap pNPTS and flank1 for *cpdR* deletion, fw | CTGCAGGATATCTGGATCCAAGCTTGCGGCCATAGATCGG |
| OKH58 | overlap pNPTS and flank2 for *cpdR* deletion, rv | CCTGTACATCCGGAGACGCGTCGTGGTGGTGCCGATGTTCAT |
| OKH59 | overlap for rif deletion of *cpdR* flank1, rv | GCCGGGACTCTGGGGTTCGAGGGGTCACGTGTCCGATTAA |
| OKH60 | overlap for rif deletion of *cpdR* flank2, fw | GTTTTATCTGTTGTTTGTCGTCCCACAAAAAGTTTCGACA |
| OKH64 | amplify *rif* cassette, rv | GCACCTTGTCGCCTTGCGTATAATC |
| OKH71 | gibson pBXMCS-2 + *cckA* fw | CTCGAGTTTTGGGGAGACGACCATATGGCCGACTTGCAGCTCCA |
| OKH72 | gibson pBXMCS-2 + *cckA* rv | GGCCGCTCTAGAACTAGTGGATCCCCCGGGCTCTACGCCGCCTGCAGCTGCTG |
| OKH79 | qChIP primer for CtrA boxCD, fw | CATGGGGTTAACGCTCTGTT |
| OKH80 | qChIP primer for CtrA boxCD, rv | CTGTCGTGTCTCAGGACGTT |
| OFS285 | pNPTS138_rev_GA | TGGATCCAGATATCCTGCAGAGAAGC |
| OFS286 | pNPTS138_fw_GA | GACGCGTCTCCGGATGTACAGG |
| OFS321 | pBXMCS_GA_rev | ATGGTCGTCTCCCCAAAACTCGAG |
| OFS360 | pB/R(V/X)MCS-2_GA_fw_new_compatible | AGCCCGGGGGATCCACTAGTTCTAGAGCGGCC |

**S2 Table. Sequences of the primers used in this study.**
